# Health system in Yemen close to collapse

**DOI:** 10.2471/BLT.15.021015

**Published:** 2015-10-01

## Abstract

Yemen is facing a growing humanitarian catastrophe as health workers there risk their lives to help civilians caught up in the deadly conflict. Dale Gavlak reports.

Since armed conflict erupted on 19 March, Yemen’s already fragile health system has come under enormous strain. 

The emergency health-care needs of the population have now become so great that health workers are struggling to provide essential health care.

 “The health system is on the brink of collapse,” says Dr Ahmed Shadoul, the World Health Organization (WHO) Representative for Yemen.

WHO is coordinating the humanitarian response to health issues with Yemen’s Ministry of Health and 20 partner humanitarian organizations in Yemen, including the International Committee of the Red Cross (ICRC) and Médecins Sans Frontières (MSF).

Heavy bombardment and airstrikes combined with continuous fighting, with few ceasefires allowing for humanitarian activity, have hampered citizens’ access to health care and increased the pressure on the health facilities that are still functioning.

“Right now everybody – international and Yemeni health workers – is focusing on emergency health provision because of the massive numbers of war wounded,” says ICRC health coordinator for Yemen, Monica Arpagaus.

The fighting has reached 21 of the country’s 22 governorates (provinces) and more than 4000 people have been killed since March in this impoverished country at the tip of the Arabian Peninsula, according to the United Nations. 

“WHO is committed to ensuring that all Yemenis continue to have access to health services, including those in the hardest-to-access areas,” says Shadoul, “through the provision of emergency life-saving medicines, trauma kits, interagency emergency health kits, diarrhoeal disease kits and blood bank supplies which are urgently needed.”

People in several governorates are suffering particularly from a lack of health care due to the fighting, such as Taiz in the south and Sa’ada in the north, Shadoul says. 

When fighting intensified in Taiz in late August and early September, there were no formal rescue services so residents had to dig out their loved ones from the rubble of damaged buildings and bring the injured to the hospitals themselves. 

“When the warring parties were both present in Taiz, it was impossible for us to pick up the wounded because neither side would stop shooting when the ambulances attempted to move,” says Hassan Boucenine, head of the MSF Yemen Mission. 

Intense fighting in Sa’ada resulted in the destruction of the ICRC Maran health clinic. “There were 18 airstrikes in Sa’ada during the first week of September,” Boucenine recalls. “It was unbelievable. The team [of health workers] had to hide inside the hospital.” 

Meanwhile access to health services is deteriorating in other parts of the country too, including Hodeida and Hajjah governorates, where most of the internally displaced have fled, as well as in most others including Hadramout, Aldhaleh, and Abyan, Shadoul notes.

In addition to restricted access to health facilities there is a severe shortage of medical supplies and equipment and Yemen’s health system is largely dependent on what WHO and its humanitarian partners can bring into the country, “but these supplies won’t be able to cover all the gaps,” Shadoul says.

The ICRC and WHO are delivering water in many parts of Yemen. WHO is supplying fuel to hospitals across the country to keep electricity generators functioning for operating theatres and for the country’s blood banks and labs, as well as petrol for ambulances. Shadoul says: “WHO has provided the entire fleet of ambulances with geographic positioning systems and is sponsoring the operational cost of more than 15 ambulances.” 

Since the crisis in Yemen escalated in March, health facilities have been hit by bombs and health and humanitarian workers are increasingly targeted.

“Almost 23% of the health facilities in Yemen are no longer functional.”

Ahmed Shadoul

“Almost 23% of the health facilities in Yemen are no longer functional either because they were hit, they were already in poor condition or they happened to be close to military targets,” Shadoul says, adding that many health workers and patients are too afraid to come to the health facilities. 

“We are getting shot at in our cars. I was almost killed by an airstrike,” said MSF’s Boucenine.

On 2 September, a gunman opened fire on an ICRC vehicle killing two Yemeni staff as they travelled through the northern province of Amran. The killings come just over a week after gunmen raided the ICRC's offices in the city of Aden.

“The deaths of our colleagues remind us of the risks we take every day in countries in conflict.”

Monica Arpagaus

“The deaths of our colleagues remind us of the risks we take every day in countries in conflict,” says Arpagaus of the ICRC, which has been supporting 19 primary health centres and nine hospitals throughout Yemen.

After visiting the country in August, the president of the ICRC Peter Maurer remarked that "Yemen after five months [of fighting] looks like [the] Syria[n Arab Republic] after five years." 

According to the United Nations, more than 1.4 million people have been internally displaced and almost 80% of the population – 21 million since March – requires some form of humanitarian assistance. An estimated 7 million people, almost one third of the population, face hunger. 

“The whole population may soon need humanitarian help,” says Boucenine, pointing to the arms embargo mandated by the United Nations Security Council to stop weapon deliveries to the rebels. The resulting inspections of all imports means that commercial and humanitarian shipments by sea and air into Yemen are severely restricted.

“How can a country that imports 90% of its food and 100% of its drugs and that can only access a small portion of these not be in trouble?” Boucenine asks.

Shadoul recalls the deep concerns at WHO when the current conflict started on 19 March since the country’s health system was already weak. 

The United Nations promptly set up the health cluster, a system of creating a group of United Nations agencies responding to a particular aspect of an emergency. WHO, as the United Nations health cluster lead, hosted a meeting of humanitarian and health organizations in the capital, Sana’a.

 “In the first 10 days of the crisis, we distributed supplies to different areas including Sa’ada, Hodeida and Mukalla.”

“We delivered further supplies to other areas and started searching for emergency hospital staff across the country, especially anaesthetists and operating theatre assistants, to treat the injuries,” Shadoul adds.

About 65 WHO national staff immediately set about working with partner organizations to distribute the medical supplies and treatment, including trauma kits for surgery. WHO is also maintaining the national surveillance system for identifying and responding to outbreaks. 

Since March of this year, the Saudi-led 10-member coalition of Gulf states has battled Houthi rebels vying for control over Yemen and the conflict has shown no signs of letting up.

At the end of March following three nights of Saudi-led airstrikes, the United Nations evacuated most of its 100 international staff. About 250 other foreigners working for nongovernmental organizations and companies also left the country. 

On 29 March, the United Nations health cluster meetings resumed in the nearby Jordanian capital of Amman. Shortly afterwards, Shadoul joined the first senior humanitarian mission to Yemen. 

In the capital Sana’a, he and his team negotiated the first humanitarian pause or ceasefire in May so that essential health care and vaccines could be delivered. 

Recent outbreaks of malaria and dengue were unusually large, health officials say, because tap-water supplies have been disrupted and people are collecting water in containers, creating breeding grounds for mosquitoes. The distribution of mosquito nets and insecticide sprays has been hampered due to the lack of security.

After more than 6000 cases of dengue fever were reported in Aden, Hodeida and other governorates in June, the outbreak was brought under control, while a sharp increase in dengue cases was reported in Taiz early last month, Shadoul says. 

WHO recently launched a nationwide campaign against polio and measles with the United Nations Children’s Fund and the Global Alliance for Vaccines and Immunization. About 4.5 million children under the age of five were immunized against polio and 6 million children under 15 years against measles. 

A second campaign is needed but has not been possible due to the fighting.

WHO estimates that about US$ 151 million is needed in funding for the health cluster’s work until December 2015, including US$ 105 million for WHO’s activities. So far only a fraction of that, US$ 20 million, has been received.

**Figure:**
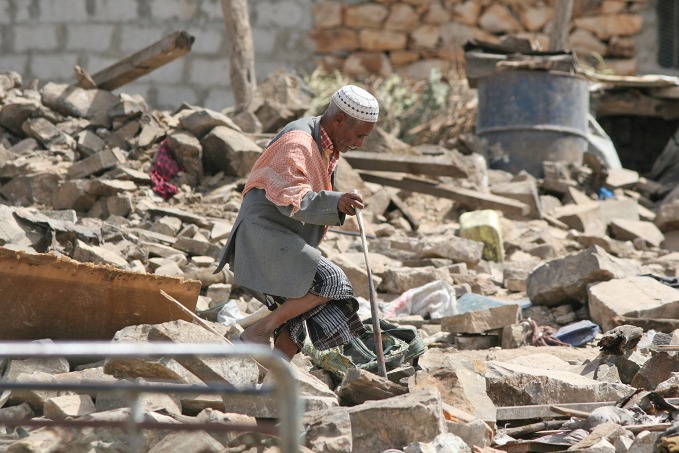
Elderly man walks through the rubble in Taiz following bombardments.

ICRC/Anees Mahyoub

**Figure:**
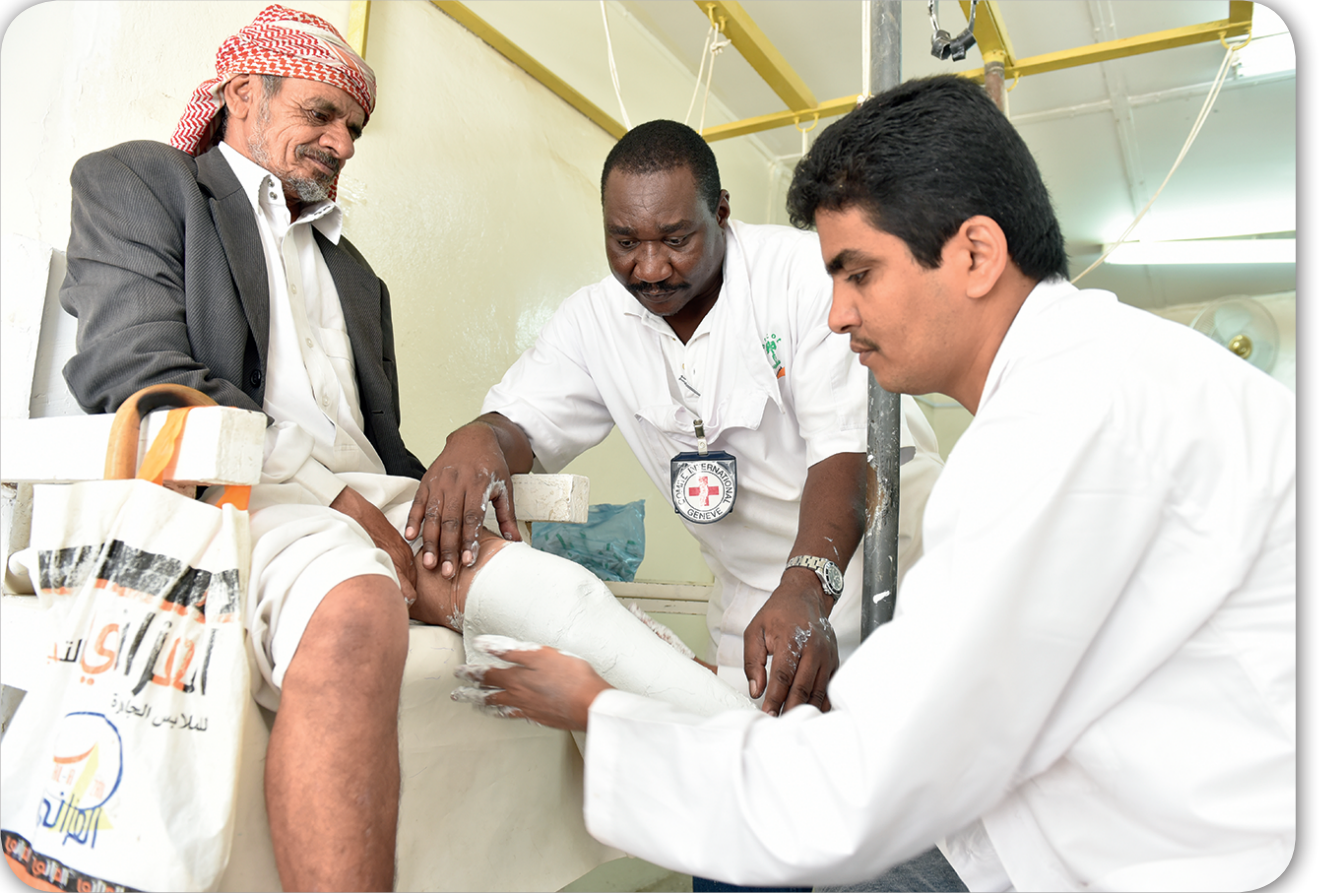
ICRC staff treat a leg injury in the city of Taiz.

ICRC/Shahin Ammane

